# Neurological Insights: Transient Unilateral Relative Afferent Pupillary Defect in the Context of Serotonin Syndrome

**DOI:** 10.7759/cureus.75321

**Published:** 2024-12-08

**Authors:** Jordanne Nichols, Konner Feldhus

**Affiliations:** 1 Biomedical Sciences, Rocky Vista University College of Osteopathic Medicine, Parker, USA; 2 Internal Medicine, Saint Joseph Hospital-Intermountain Health, Denver, USA

**Keywords:** afferent pupillary defect, polypharmacy, polypharmacy overdose, serotonin, serotonin syndrome

## Abstract

The pupillary reflex is a complex physiological response involving both the eye and associated neural pathways. Relative afferent pupillary defects (RAPDs) can present due to various underlying pathologies, yet their occurrence in serotonin syndrome has been rarely documented. This report details the case of a 21-year-old non-binary individual with a history of bipolar disorder who presented to the emergency department following a polypharmacy overdose, including significant ingestion of sertraline, aripiprazole, and buspirone. The patient exhibited symptoms consistent with serotonin syndrome, prompting immediate intervention. Notably, a unilateral afferent pupillary defect was identified alongside mydriasis, hyperreflexia, and inducible clonus. This case highlights the significance of clarifying the connection between RAPD and serotonin syndrome to improve clinical recognition and understanding of how excess serotonin affects neurological and ophthalmic processes.

## Introduction

The pupillary reflex is a complex process involving the physiology of the eye and related brain structures. Direct response occurs when light is directed at one eye, leading to immediate constriction of that pupil, while consensual response refers to the constriction of the opposite pupil due to the neural pathways involved [1]. Afferent dysfunction of the pupillary reflex, known as a relative afferent pupillary defect (RAPD), may be clinically observed when the direct response in one eye is either unresponsive or unequal compared to the constriction of the contralateral eye [1]. Although afferent pupillary defects are linked to various pathologies in the literature, they have rarely been documented in the context of serotonin syndrome. Serotonin syndrome itself is the clinical manifestation of excessive serotonergic activity, typically resulting from the build-up of centrally acting agents that elevate serotonin levels [2]. Several criteria have been established for diagnosing serotonin syndrome. The diagnosis generally requires recent use of serotonergic agents along with the presence of autonomic instability, such as hyperthermia, tremor, clonus (either spontaneous or inducible), hyperreflexia, or hypertonia [2]. Management involves discontinuing the serotonergic agent and providing supportive care, including intravenous fluids and treatment of clinically apparent autonomic instability [2]. This case describes a young adult with an intentional polypharmacy drug overdose leading to serotonin syndrome, accompanied by the finding of an RAPD.

## Case presentation

A 21-year-old non-binary individual with a past medical history of alcohol use disorder and bipolar Ⅰ disorder presented to the emergency room after an attempted suicide. Prior to the suicide attempt, the patient’s home medications included 12.5 mg of aripiprazole and 25 mg of buspirone. Suicide was attempted by ingesting roughly 40 tablets of 50 mg sertraline, seven tablets of 12.5 mg aripiprazole, and seven tablets of 25 mg buspirone while also consuming alcohol. The blood alcohol level at the time of admission was 149 with a positive urine drug screen for tetrahydrocannabinol (THC). The patient on admission was vomiting, agitated, tachycardic, afebrile, and normotensive. The patient was alert and oriented to person, place, and time. A basic metabolic panel and complete blood count done on admission were within normal limits. However, liver function tests revealed an elevated total bilirubin of 1.8 mg/dL with a direct bilirubin of 1.5 mg/dL. The patient was administered 1 L of intravenous fluids in the emergency department and remained on room air. Due to the extreme risk of serotonin syndrome, the patient was given 1 mg of midazolam subcutaneously and was admitted under suicide precautions to the telemetry unit. Suicide precautions included the use of a 24-hour patient sitter in addition to a bed alarm. Poison control was contacted and consulted to ensure appropriate care was provided.

On the morning after admission, the patient was noted to have bilateral mydriasis with an afferent pupillary defect on the left but an intact afferent pupillary reflex on the right. All other cranial nerves were intact at the time of the exam, in addition to symmetric strength testing in the upper and lower extremities. The patient was noted to have 3+ reflexes in the upper and lower extremities with bilateral inducible clonus of the ankles, consistent with serotonin syndrome. An orbital MRI was discussed with an ophthalmology consult, who recommended outpatient follow-up for the RAPD if there was incomplete resolution in association with the other symptoms of serotonin syndrome. A final examination later that day, 24 hours after the polypharmacy ingestion, revealed the resolution of the afferent pupillary defect, confirming it to be transient. Due to the improvement of symptoms, the orbital MRI was canceled. Care was then transferred to inpatient psychiatry, where the patient continued to do well and was safely restarted on aripiprazole in addition to lithium.

## Discussion

Polypharmacy drug overdose presents significant complexity due to the potential for drug interactions and the challenge of evaluating symptoms with unclear origins. In this case of intentional overdose, the patient ingested a substantial amount of sertraline, alongside buspirone and aripiprazole. Sertraline, a selective serotonin reuptake inhibitor (SSRI), works by inhibiting the reuptake of serotonin into the presynaptic neuron, thereby increasing the availability of serotonin for neurotransmission [3]. Buspirone acts as a partial agonist at the 5HT1 serotonin receptor, which has a partially similar mechanism of action to the antipsychotic aripiprazole, a partial agonist at both the 5HT1a serotonin receptor and D2 dopamine receptor [4,5]. Sertraline and buspirone both independently pose risks of serotonin syndrome due to their perpetuated mechanisms of action on serotonin and its associated receptors [2-4]. When taken in large quantities, as seen in this case, these medications can significantly increase the risk of developing autonomic instability due to excess serotonin, resulting in the clinical diagnosis of serotonin syndrome.

The management of serotonin syndrome primarily involves the discontinuation of any medications that either increase serotonin levels or enhance its effects [2]. In cases where symptoms are severe or rapidly progressing, sedatives such as benzodiazepines, like midazolam, are administered to control agitation and prevent dangerous outcomes like seizures [2]. In situations where initial interventions do not resolve symptoms or if the patient remains at risk for further deterioration, pharmacologic antidotes such as cyproheptadine may be considered [2]. Cyproheptadine is a serotonin antagonist that can help counteract serotonin’s effects, but its use is typically reserved for cases where sedative approaches and supportive care are insufficient [2]. In this case, however, the patient's symptoms were managed effectively with sedative treatment, and there was no need for cyproheptadine administration. Imaging is generally not included in the standard criteria for diagnosing serotonin syndrome, which is why neuroimaging was not initially ordered for this patient [2]. An orbital MRI was considered only after the RAPD raised concerns for further evaluation, although it was canceled after the clinical resolution of symptoms.

Mydriasis and clonus are common ocular findings in serotonin syndrome, both of which contribute to the clinical presentation necessary for diagnosis [1,2]. Mydriasis (pupil dilation) results from serotonin’s influence on autonomic overactivity, due to excess sympathetic stimulation, while clonus arises from the hyperexcitability of the neuromuscular junction, leading to rhythmic, involuntary muscle contractions that can occur spontaneously or in response to external stimulation [2]. Although this patient presented with pronounced mydriasis as a manifestation of serotonin syndrome, an abnormal response to light stimulation would not typically be anticipated. Diminished or absent consensual pupillary constriction when light is shined into one eye typically suggests a disruption in the afferent limb of the pupillary light reflex pathway [1]. The afferent limb of the pupillary light reflex involves the retina, optic nerve, optic chiasm, and the pretectal nucleus in the midbrain, which communicates with the Edinger-Westphal nucleus to mediate pupillary constriction via parasympathetic fibers traveling through the oculomotor nerve [1,6]. Intact visual acuity, as seen in this case, suggests that the acute lesion did not significantly affect the central visual pathways, such as the optic nerves or primary visual cortex, but rather affected the afferent input at a higher level in the brain [1,6]. This is because lesions at or distal to the optic chiasm, including those in the optic tract or pretectal area, can lead to isolated deficits in the pupillary reflex pathway without causing a corresponding loss in visual acuity [1]. Current literature on the specific effects of serotonin on structures such as the optic tract and pretectal area is limited, and further research is needed to elucidate the underlying physiological mechanisms. Investigating this relationship could enhance our understanding of visual processing and the broader implications of serotonin dysregulation in neurological conditions. Furthermore, innovative education on the link between transient RAPD and serotonin syndrome could help prevent the unneeded ordering of neuroimaging, to avoid unnecessary costs for both hospitals and patients. However, due to the rare prevalence of transient unilateral RAPDs in the literature and its unknown mechanism, neuroimaging may still be warranted if the defect persists after the clinical symptoms of serotonin syndrome have resolved.

## Conclusions

Transient unilateral RAPD, as observed in this case, is a rare finding in the literature in association with serotonin syndrome. Acknowledging this correlation may contribute to a reduction in unnecessary neuroimaging procedures, benefiting both patients and healthcare systems. Further research is essential to evaluate the prevalence of RAPD in serotonin syndrome and to clarify the mechanisms by which serotonin influences pupillary responses.
